# Acceptability of the content and functionality of a just-in-time adaptive intervention for gambling problems: a mixed-methods evaluation of gambling habit hacker

**DOI:** 10.1186/s13722-025-00573-y

**Published:** 2025-06-05

**Authors:** S. N. Rodda, S. S. Merkouris, C. J. Greenwood, A. C. Thomas, D. I. Lubman, N. A. Dowling

**Affiliations:** 1 Department of Psychology and Neuroscience, Auckland University of Technology, 90 Akoranga Drive, Auckland, New Zealand; 2 School of Psychology, Deakin University, Burwood, Australia; 3 Monash Addiction Research Centre and Eastern Health Clinical School, Faculty of Medicine, Nursing and Health Sciences, Monash University, Melbourne, VIC Australia; 4 Turning Point, Eastern Health, Melbourne, VIC Australia

**Keywords:** mHealth, Implementation planning, Harm reduction, Ecological momentary intervention, Goal setting, Treatment

## Abstract

**Background:**

Just-In-Time Adaptive Interventions (JITAIs) offer real-time support to help individuals adhere to gambling expenditure goals. This mixed-methods study evaluated the acceptability of a JITAI called *Gambling Habit Hacker*, focusing on both the app’s content and functionality.

**Methods:**

Australian participants who had recently completed a micro-randomized trial of the app provided feedback through semi-structured interviews. Acceptability was further assessed using app usage and engagement metrics, including registration, adherence to the ecological momentary assessment (EMA) protocol, duration and timing of app use, and interaction with intervention content. Subscales of the Mobile App Rating Scale (MARS) were also used. Intervention fidelity was evaluated by examining adherence to the EMA protocol, strategy selection, and action plan completion.

**Results:**

Participants (*n* = 174) completed 4382 EMA entries over a 28-day period, averaging 25 EMAs each, with the highest completion rate observed in Week 1. Of those, 48% were micro-randomized to receive the intervention, and 80% of this group completed at least one intervention. Across the trial, participants created 1307 action and coping plans, most commonly focused on behaviour substitution, initiating rewards, or limiting access to cash. In the post-intervention survey (*n* = 141), participants reported increased awareness, knowledge, and shifts in attitudes toward gambling, exceeding minimally acceptable thresholds. Semi-structured interviews with 11 participants revealed two primary motivations for using the app: (1) goal setting with monitoring, and (2) goal adherence with an emphasis on managing urges. Qualitative and quantitative findings suggest future optimisation should include customising the timing and frequency of EMAs and adopting a hybrid push–pull approach to intervention delivery. Functionality improvements could also include the ability to save and adjust action plans in real time to enhance responsiveness in high-risk situations. Engagement may be further improved by incorporating additional lived experience content, such as videos and audio recordings.

**Conclusion:**

*Gambling Habit Hacker* was found to be acceptable for providing support in adhering to gambling expenditure limits and, over time, reducing gambling spend. Future optimisation could improve its tailoring to individual needs and enhance user engagement.

**Trial registration:**

This trial was registered with the Australian New Zealand Clinical Trials Registry (ACTRN12622000497707) and approved by the Deakin University Human Research Ethics Committee (2020 − 304).

**Supplementary Information:**

The online version contains supplementary material available at 10.1186/s13722-025-00573-y.

## Background

In-person treatments for gambling problems, such as Cognitive Behavioural Therapy (CBT) and Motivational Interviewing, are effective for those who can access them [1, 2]. However, only one in five people with problem gambling seek any form of help, and this drops to one in 25 for those at moderate risk [3]. To address these low rates of help-seeking, alternative treatment modalities have emerged. A review of all randomised controlled trials (RCTs) of psychological gambling interventions found that 24% were self-help, with 26% of those delivered online [4]. A recent review highlighted that most evidence supports internet-delivered CBT that mirrors traditional counselling formats, such as weekly sessions over six weeks. Other digital options—like email and chat counselling, self-help tools, online forums, and smartphone apps—are also popular, though the evidence base for these is generally weaker [5]. Web-based treatments are appealing because they offer a convenient, accessible, and low-cost way to access support anytime and from anywhere [5, 6].

In recent years, health-related interventions delivered via smartphones or mobile devices—referred to as mHealth—have become a popular way to monitor and tobacco, alcohol and other drug use [7]. In the context of gambling problems, mHealth can provide psychological treatments such as Cognitive Behavioural Therapy (CBT) and mindfulness, as well as tools for self-monitoring gambling expenditure, budgeting, mood tracking, and goal progress. Apps may also include quizzes on the signs of gambling problems, educational content, community support features, and information about blocking software [8–11]. Gambling support apps are typically free, and public attitudes toward them are generally positive [12]. However, many publicly available apps receive low satisfaction ratings and often lack formal evaluation [8], with most supporting abstinence-based goals [10, 11]. These apps can be either static— delivering the same content to all users—or dynamic—tailoring content to individual needs [8]. Dynamic apps aim to deliver the right support at the right time by adapting interventions to users’ current circumstances. Just-in-Time Adaptive Interventions (JITAIs) use real-time data collection, known as Ecological Momentary Assessments (EMAs), to assess when and what type of support is needed [13–15]. These dynamic apps can engage users either through push mechanisms, where the app initiates contact via notifications, or pull mechanisms, where users initiate engagement when they recognise a need for support.

Because mHealth is an emerging area of research, much of the focus is on acceptability as part of intervention development and evaluation. The acceptability of an intervention has recently been operationalised as a multidimensional construct that reflects its perceived appropriateness based on cognitive and emotional responses to the intervention [16]. A focus on acceptability ensures that new interventions are delivered in an appealing form, and that the content of interventions is relevant and seen as worthwhile to maintain engagement. Although it is an emerging field, there have already been studies examining the acceptability for mHealth for gambling problems which indicate positive attitudes towards mHealth but low engagement with study protocols [11, 17–20]. These findings highlight strong interest in mHealth but very limited knowledge of what might work for people with gambling problems.

### The development and functionality of gambling habit hacker

**Fig. 1:**
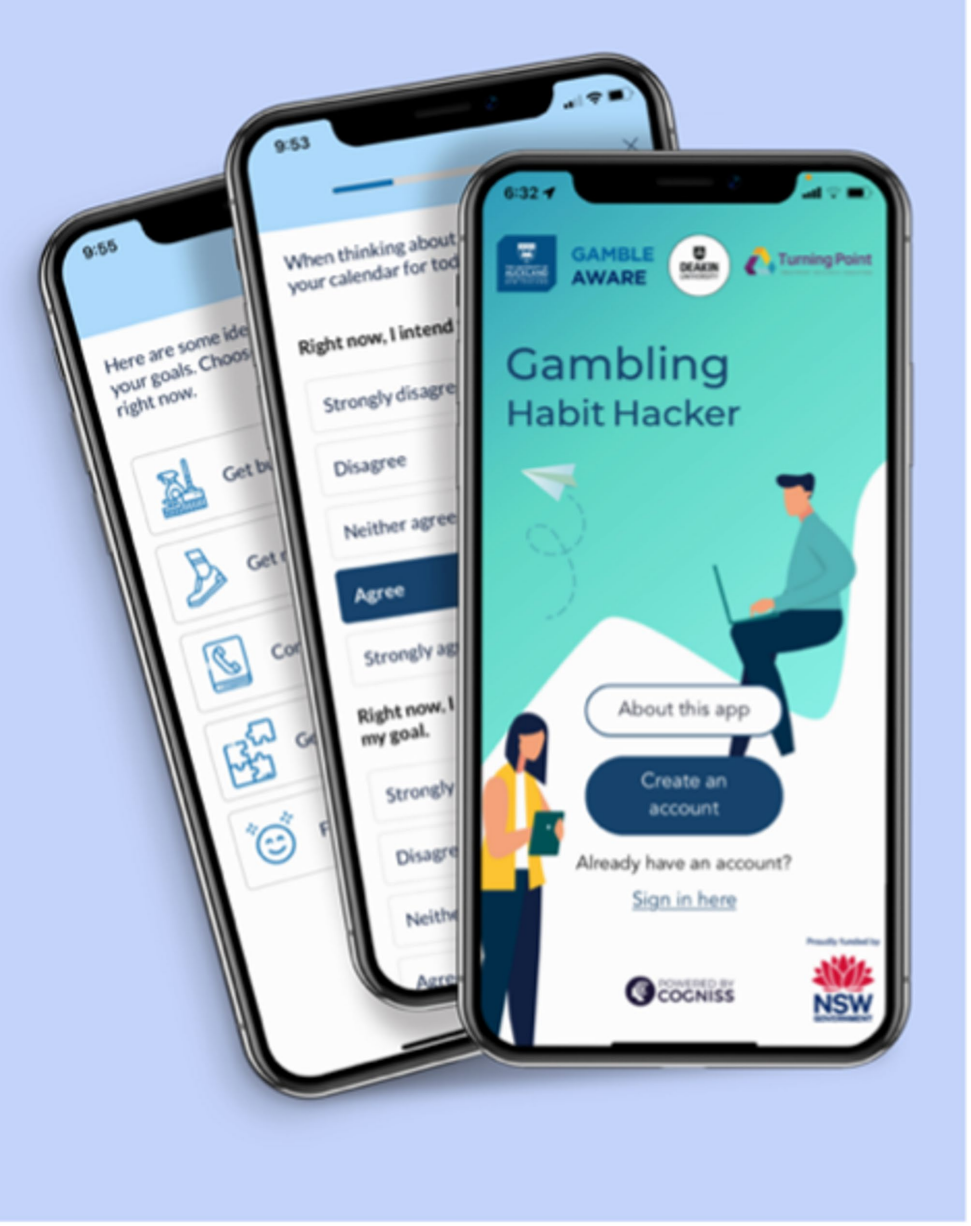
Illustrative gambling habit hacker screenshots

This study aimed to conduct a comprehensive evaluation of *Gambling Habit Hacker*, a new, theoretically informed dynamic app designed to provide real-time support for adhering to gambling expenditure goals [21, 22]. Gambling Habit Hacker is a JITAI that “pushes” the right support at the right time, in a form and amount tailored to individual needs (see Fig. 1 for illustrative screenshots). The app is intended for individuals seeking to reduce their gambling and provides practical assistance for sticking to self-set expenditure limits. It is grounded in the Health Action Process Approach [23, 24] and responds to empirical evidence highlighting a gap between the intention to reduce gambling expenditure and actual behaviour [25–30]. The long-term (distal) aim of the app is to reduce gambling expenditure, while the short-term (primary proximal) goal is to improve adherence to gambling expenditure plans. Secondary proximal outcomes include strengthening goal intention, goal self-efficacy, urge self-efficacy, and the ability to manage high-risk situations. The app is delivered in line with self-determination theory [31], which emphasises the individual’s autonomy in setting personal goals and offers support to enhance competence and self-efficacy in achieving them.

*Gambling Habit Hacker* uses a JITAI design framework [13] which delivers tailored support when implementation assistance is needed. The app applies decision rules to target momentary fluctuations in intention strength, goal self-efficacy, urge self-efficacy, and the presence of high-risk situations. Vulnerability is detected through EMAs, administered three times daily at designated decision points. These EMAs include 18 items assessing intention strength, goal self-efficacy, and urge self-efficacy, along with 15 items measuring high-risk situations [21, 22]. When goal vulnerability is identified, the app uses these responses as tailoring variables to deliver personalised interventions aligned with the user’s current needs.

The intervention content is designed to support implementation intentions through goal setting, action planning, coping planning, and self-monitoring, and includes extensive support for self-enactable behaviour change strategies [21, 22]. Goal setting is guided by the Timeline Follow Forward method—a calendar-based system in which participants plot their intended gambling activities across the 28-day intervention period [32]. Each participant’s goal is saved on their home page, along with their planned gambling days, times, and total intended expenditure. This process is supported by information on Australia’s lower-risk gambling guidelines, which recommend gambling no more than three times per month and limiting spending to $50 per month [33]. Action and coping planning are carried out within the app, with strategies tailored to the user’s current level of goal vulnerability. Based on these tailoring variables, the app delivers a set of relevant strategies (*n* = 120), each with detailed implementation guidance (see protocol paper for specific algorithms [21, 22]). Participants choose a strategy in the moment—such as from the “do something else” group, which includes options like “Get busy,” “Get moving,” “Make a connection,” “Get a positive addiction,” and “Feel good.” Each strategy presents 50–100 words of lived-experience-based guidance [26–30], quotes, and suggestions, spread across 4–5 screens. After reviewing the material, participants are prompted to create their own implementation plan in the form of an action plan and a coping plan.

Preliminary usability testing was conducted with 14 gambling experts during the formative stages of app development [22]. This group included individuals with lived experience of problem gambling, gambling counsellors, and researchers, who used the app over a three-day period and provided both quantitative and qualitative feedback on its usability, acceptability, and overall quality. The app received an overall quality score of 3.43 on the Mobile App Rating Scale (MARS) [20], with particularly high ratings for its planning functionality, implementation support, and perceived impact. The highest-rated impact areas included increasing awareness, knowledge, and help-seeking, followed by influencing intention, attitudes, and behaviour change. Based on feedback, several lower-rated aspects were improved, including the functionality of the goal-setting calendar, the length of baseline and EMA items, and the timing of notifications. In line with literature on implementation support [23, 34–36], the app was originally designed to deliver coping planning 30 min after action planning. However, user testing revealed this approach was confusing and burdensome, leading to the two being integrated into a single streamlined intervention delivered simultaneously.

*Gambling Habit Hacker* was recently evaluated through a micro-randomized trial (MRT) and a within-group six-month follow-up study [37]. The MRT employed a sequential factorial design in which each participant served as their own control. Each time an Ecological Momentary Assessment (EMA) identified a participant as eligible; the app randomly assigned them to receive either an intervention or a control condition. When randomised to an intervention, participants were prompted to select from a range of 25 strategy groups that aligned to 10 BCT categories. Although the MRT found no significant difference between intervention and control conditions on proximal adherence to gambling expenditure goals, there were significant improvements in overall gambling expenditure, gambling symptom severity, situational self-efficacy, psychological distress, and well-being at post-treatment—effects that were maintained at the six-month evaluation. This trial was the first to evaluate a JITAI for gambling problems in individuals with less severe gambling issues, using methods other than Cognitive Behavioural Therapy (CBT). However, the study faced several limitations, including difficulties in accurately measuring goal adherence and low participant engagement. EMA compliance was particularly low, with only 30% of prompts completed, despite the offer of additional reimbursement for higher completion rates. Participants were compensated with small payments of AUD $1 for each completed EMA, and those who completed more than 75% of EMAs received a bonus of AUD $20, with total compensation capped at AUD $100.

### Current study aims

The present study aimed to assess the acceptability of *Gambling Habit Hacker* in participants who participated in the MRT [37]. This mixed-methods investigation evaluated the app’s acceptability using app usage and engagement metrics, post-intervention survey responses collected 28 days after registration, and semi-structured interviews conducted after participants had completed their use of the app. Intervention fidelity was examined through adherence to the EMA protocol, strategy selection, and completion of action plans. The mixed-methods design enabled a comprehensive understanding of both the extent of participant engagement (quantitative) and the reasons behind that engagement (qualitative). A phenomenological approach guided the qualitative component, focusing on the lived experiences of individuals who used *Gambling Habit Hacker*. Insights from this study offer valuable guidance for optimising the app and contribute to the broader development of limit-setting tools and mobile health interventions for gambling and other behavioural addictions.

## Methods

### Study design

A concurrent mixed-methods approach was used, with data collected during the 28-day MRT period and the six-month follow-up. The acceptability evaluation included app usage and engagement metrics from all 174 MRT participants during both the MRT and follow-up periods; app acceptability measures collected through a post-intervention survey (*n* = 141); and semi-structured interviews conducted 1–3 months after using *Gambling Habit Hacker* (*n* = 11). Quantitative and qualitative findings are presented separately, followed by an integrated synthesis in the discussion section. Reporting was informed by the Mixed Methods Appraisal Tool [38] as well as the Consolidated criteria for Reporting Qualitative Research (COREQ) for interviews and focus groups [39].

### Participants and procedure

The study was conducted in Australia, where gambling is legal and widely available across poker machines, wagering, sports betting, and casinos. Each state and territory funds free treatment and support services, accessible in person or by phone at no cost [40]. These are complemented by websites offering information, screening tools, self-help resources, and links to further treatment. Participants in the quantitative component of this study were individuals who had registered for *Gambling Habit Hacker* and met the minimum inclusion criterion of completing at least one EMA during the 28-day trial period. Additional eligibility criteria included: (i) current residency in Australia, (ii) age 18 years or older, (iii) installation of the app on a personal smartphone with internet access, (iv) willingness to allow notifications, (v) fluency in English, and (vi) a desire for support with gambling. There was no requirement to meet diagnostic criteria, and participants were free to seek other forms of help.

*Gambling Habit Hacker* is available on both the Google Play and iOS app stores. It was a free app for Australian residents, promoted to those wanting to “change your gambling habits” and “discover your personal strengths, build confidence, and get motivated.” To join, users provided consent, agreed to terms and conditions, created an account (username, email, password, display name), indicated interest in an optional interview, and completed a pre-intervention survey, with account verification via email and a plain language statement attached. After reviewing the app description and instructions, participants completed a brief survey, provided a mobile number, and indicated interest in post-treatment interviews. Participants then began using the app, starting with their first EMA, with all assessments conducted in-app and no in-person contact required. By accepting the terms, participants confirmed they understood the study, met eligibility criteria, and consented to take part, with developer details and affiliated institution logos displayed on the app’s landing page. Recruitment ran from April to December 2022 through convenience sampling, using channels such as social media, gambling-related websites (including government sites), public notices, counselling services, gambling venues, and word of mouth.

A total of 141 participants completed the post-intervention survey and measures of app acceptability. Participants were sent an email one week before the six-month follow-up survey was due. If the survey was not completed, reminder protocols were implemented: an initial reminder email was sent one week after the due date, followed by up to two additional reminders via phone, text message, or email over the following three weeks. Participants who did not complete the post-intervention survey were still invited to complete the six-month follow-up. Those who completed both the post-intervention and follow-up surveys received AUD$50 in remuneration.

Eleven participants were recruited from this sample to complete semi-structured interviews. Interest in participating in the interviews was indicated in the pre-intervention survey. Purposeful sampling was used to ensure representation across gender and levels of app usage (categorised into tertiles based on the first round of micro-randomised trial recruitment), as well as to ensure adequate representation of individuals residing in New South Wales (in alignment with the funding source). Participants who completed the interview received an additional AUD$30 e-gift voucher. Of the interview sample, seven participants were male. The group represented a range of gambling behaviours (see Supplementary File S2). Seven participants aimed to reduce their gambling expenditure, while four intended to maintain their current spending levels. Among the latter, three had already aligned their gambling with recommended guidelines (i.e., $50 per month), while one participant reported a planned spend of $140 per month. App usage varied, with EMA compliance categorised as low (4–8 entries), moderate (9–58), or high (59 or more) over the 28-day period.

### Measures

#### App usage and engagement indices

App use and engagement data was collected for the entire sample across the 28-day MRT period using data retained in the *Gambling Habit Hacker* app. All app use and engagement was measured directly from the app and included data relating to registration in the app, engagement with the EMA protocol, app use duration, timing of app use, and engagement with the intervention. Post-MRT app use and engagement indices were also examined.

#### Measures of app acceptability

The post-intervention assessment of acceptability involved participants’ quantitative evaluation of the app in terms of subjective quality, perceived impact, and potential areas for optimisation. The survey was administered using Qualtrics survey software, with participants receiving an email containing a link to complete the assessment. The survey included both post-treatment metrics and items specific to the app’s acceptability.

The Mobile App Rating Scale (MARS) [20] was used to assess subjective quality (4-item subscale, variable 5-point scale), including items such as likelihood of recommending the app to others and an overall star rating. The MARS also assessed perceived impact using a 6-item subscale rated on a 5-point agreement scale, capturing outcomes such as increased awareness, knowledge, and changes in attitudes. MARS is a widely recognised and validated tool for assessing app quality [41], with subscale scores of 3 or higher indicating minimum acceptability [42].

Additional feedback was gathered on support and accessibility through 16 items addressing who could provide support (e.g., counsellor, individual with lived experience, or coach), preferred support modalities (e.g., text, chat, email, phone, video, or in-person), and preferences for app features. Participants rated the perceived helpfulness of potential enhancements such as discussion boards, on-demand access, motivational messages, a virtual AI coach, feedback on gambling progress, in-app rewards, and the ability to save favourite features. Each item was rated on a 5-point scale ranging from “not at all helpful” to “very helpful.” Participants were also asked about preferences for the frequency of EMA delivery, program duration, and mode of intervention delivery (push notifications, pull-only use, or a combination of both). The survey concluded with an open-ended item inviting participants to provide any additional feedback or suggestions for improvement.

#### Semi-structured interviews

An interview guide was developed to explore participants’ experiences with *Gambling Habit Hacker*. The guide focused on both the functionality and content of the app, with questions structured around key stages of engagement, including selecting, downloading, and registering for the app. Participants were first asked about the features that initially attracted them to the app. Subsequent prompts explored goal setting, including how participants selected their goals and the technical aspects of entering goals into the app calendar. The interview schedule also included questions about participants’ experiences with the EMA protocol, such as whether the number of check-ins felt appropriate, and how they tracked their gambling expenditure against set goals.

Participants were invited to reflect on their overall experience of using the app, including components such as planning, identifying personal strengths, and completing the visualisation activity. The interview also included a section on implementation support, focusing on the lived experience stories and quotes presented within the app. Finally, participants were asked to comment on the overall look and feel of the app and were given the opportunity to share any additional experiences or perspectives. The interview schedule was pilot tested with the research team and subsequently refined to focus more specifically on app components rather than broader recovery experiences.

Interviews were planned to last 30–45 min and were conducted via video conferencing. All interviews were conducted by one female researcher (AT), an experienced gambling researcher with a PhD and extensive qualitative interviewing expertise. The interviewer had minimal prior relationship with participants, limited to email communication to schedule the session. No non-participants were present during the interviews. All interviews were audio recorded and transcribed for analysis. The first interview was reviewed by other team members (SR and ND) to confirm that the guide was effectively capturing the intended content. Participants were also offered the opportunity to review their interview transcripts. Given the narrow focus of the study and the purposive sampling across gender, gambling severity, and app use, we anticipated that 8–10 interviews would be sufficient to meaningfully explore participants’ experiences of the app [43, 44].

### Data analyses

#### Quantitative data analysis

Quantitative data analyses were conducted in Stata 17. Descriptive statistics, including means, standard deviations, medians, interquartile ranges, frequences, and proportions, were calculated to describe the app use and engagement indices and the measures of app acceptability.

#### Qualitative data analysis

Data from the semi-structured interviews were analysed using reflexive thematic analysis with an inductive approach [45]. Two coders (SR and AT) independently reviewed two full transcripts and developed an initial set of themes. These preliminary themes were then discussed among SNR, ACT, and NAD, with consensus reached that they accurately reflected the participant data. SNR then coded the remainder of the dataset and further developed the themes. Both SNR and ACT have over 20 years of experience in gambling research and bring a neutral, non-judgemental approach to conducting qualitative work in this area. The analysis involved multiple readings of the transcripts, with initial notes used to generate codes. These codes and their annotations were then grouped into sub-themes. In some cases, items were recoded to improve alignment across sub-themes. Coding focused specifically on participants’ experiences, attitudes, and perspectives, rather than on general or evaluative statements about intervention components (e.g., planning or perceived helpfulness). To support interpretation and readability, the results were structured according to the main components of the app. This approach reflected the specificity of participants’ feedback, which tended to relate directly to discrete features such as technology, goal setting, or the frequency of EMAs in relation to self-monitoring. To validate the findings, an audit trail was used. ND reviewed the annotations from the analysis and compared them with the reported results to ensure that key themes were consistently represented. Negative case analysis was also conducted, re-examining quotes or comments that were not included in the final results to assess whether they were captured within broader themes. Illustrative quotes were selected to support the thematic findings and ensure accurate interpretation. Quotes were presented verbatim, with minor edits made only to correct grammar or improve clarity.

## Results

### App usage and engagement

The sample of 174 participants who completed the MRT was predominantly male (68%), 18 to 72 years of age (Median = 35) and of Australian ethnicity (75%) (see Supplementary File S1 for full socio-demographics). Electronic gaming machines (EGMs) were the most common type of problem gambling activity (71%), followed by racing or wagering (56%), sports or events gambling (41%), games (18%), and number games (16%). Participants indicated their volitional state as not yet translated intention into action (10%), preparation (62%), implementing action (27%), or maintaining change (1%). Gambling severity, assessed by the Gambling Symptom Assessment Scale (G-SAS) [46], was most often categorized as moderate (39%), followed by severe (35%), mild (14%), and extreme (12%). The sample exhibited high rates of past-month gambling behaviour in the pre-intervention survey, with a median of 7 times and median expenditure of $1877. After prompting for goal setting, participants aimed for a median frequency of twice a month and a median expenditure of around $100. Full data collection items and demographic details are reported in the protocol and outcome publications [22, 37].

#### Registration in the app

Data were recorded from 395 individuals during the recruitment period. Of these, 23 were excluded due to ineligibility: eight signed up as “family or friends,” eight as “other stakeholders,” one was under the age of 18, and six indicated they were not residing in Australia. The remaining 372 individuals met the eligibility criteria, corresponding to an average of 49 eligible users recruited per month. Of the eligible group, 55 participants were excluded for other reasons. These included 33 affected by a technical malfunction, four who created multiple accounts, and 18 who signed up shortly after enrolling in GamblingLess: In-the-Moment—a separate gambling support app being evaluated concurrently by the research team GamblingLess: In-the-moment [19, 21, 47]. An additional 130 participants did not complete onboarding. Of these, 66 did not complete the pre-treatment survey, and 65 did not complete the initial EMA scheduled immediately after onboarding (Day 0). Thirty-six individuals were also excluded from the analytic sample as they only completed the EMA on Day 0 and did not engage with the app during the trial period. The final analytic sample included 174 participants who completed at least one EMA during the 28-day micro-randomized trial (Days 1–28). The median time to complete the pre-treatment survey was approximately 13 min (median = 13.2, IQR = 9.23–19.4), largely due to the inclusion of the Timeline Followback and goal-setting activity using the Timeline Follow Forward.

#### Engagement with the EMA protocol

Overall, 4,382 EMA protocols were completed by the 174 participants across the 28-day MRT period, resulting in a compliance rate of 30%. Participants completed an average of 25.2 EMAs (SD = 26.8, median = 10.5, IQR = 3–47, range = 1–83) of a possible 84 EMAs across the MRT period, with 14% of participants completing only one EMA. Additionally, in terms of baseline characteristics, EMA completions were higher among females (M = 37.46) than males (M = 19.82) but were not different across age or income. On average, participants completed the EMA protocol 12.4 days of the 28-day MRT period (SD = 10.8, median = 8, IQR = 2–26, range = 1–28). Participants had the option to postpone the EMA protocol using a snooze function. On average, this feature was used less than once throughout the study period (M = 0.64, SD = 2.50; median = 0; IQR = 0–0; range = 0–37). Over one-third of participants (40%) completed more than one EMA within the allowed 2-hour period at least once (M = 1.36, SD = 2.41, median = 0, IQR = 0–2, range = 0–12). During the six-month follow-up period, in which no notifications were provided, 26 (15%) participants continued to use the app. Participants completed the EMA protocol 46 times across this period (M = 1.77, SD = 1.39, median = 1, range = 1–5). The majority of these EMAs occurred only in the first two months of the follow-up period (month 1: 85%; month 2: 4%), with the remaining EMAs occurring in the 6th month (11%).

#### App use duration

The median time to complete each EMA was less than one minute (median = 0.80, IQR = 0.53–1.20). For sessions where an intervention was delivered, participants spent a median of 1.35 min in the app (IQR = 0.75–2.55). Over the full 28-day micro-randomized trial (MRT) period, the median total time spent in the app—including EMAs and intervention activities—was approximately 34 min (median = 34.41, IQR = 6.47–121.60).

#### Timing of app use

On average, participants used the app between 8 and 9 times across each time-of-day period: morning (5:00 a.m.–12:00 p.m.), afternoon (12:00 p.m.–5:00 p.m.), and evening (5:00 p.m.–5:00 a.m.). App usage declined across the 28-day MRT period, from an average of just over 8 uses in Week 1 to just over 5 uses in Week 4. Weekly engagement also decreased over time: 98.3% of participants used the app in Week 1, compared to 62.6% in Week 2, 51.1% in Week 3, and 48.3% in Week 4 (see Supplementary File S3).

#### Engagement with the intervention

At least one intervention was delivered to 80% of participants (*n* = 140). As specified in the micro-randomization protocol, 48% of completed EMA entries triggered an intervention. Strategy groups from all ten behaviour change technique (BCT) categories were selected at least once during the study period (see Table 1). Across the intervention period, participants selected a median of four different BCT categories (IQR = 1–6; range = 1–10). Among the BCT categories, substitution activities were selected by 64% of participants. Other categories selected included maintaining momentum (54%), urge management (46%), staying in control while gambling (39%), and financial management (38%). Across the 28-day trial, participants selected a median of five BCT categories (IQR = 2–15; range = 1–39), resulting in the total selection of 1307 techniques.

**Table 1:** Participant selection of each BCT category (*n* = 174)

BCT category	Mean	SD	Med	IQR 25%	IQR 75%	Total	Percent of participants
Avoidance	1.86	1.73	1	1	2	78	30%
Financial management	2.87	2.68	2	1	4	155	38%
Maintaining momentum	2.37	2.10	2	1	3	178	54%
Managing emotions	1.38	0.71	1	1	2	33	17%
Rewards	2.00	1.61	1.5	1	2	112	40%
Substitution activities	3.59	5.03	2	1	4	323	64%
Social support	2.57	3.09	2	1	3	108	30%
Staying in control while gambling	2.48	2.13	2	1	3	134	39%
Stress management	1.54	1.14	1	1	2	43	20%
Urge management	2.23	1.62	2	1	3	143	46%

Based on participants’ EMA responses, a range of intervention strategy groups were selected from the 25 available options. All strategy groups were selected by at least one participant during the intervention period (see Table 2). The strategy group “do something else” was selected by 59% of participants at least once, with a total of 305 selections across the sample. Other strategy groups selected by over 30% of participants included “grab a treat” (38%), “limit cash” (34%), “control gambling urges” (31%), “strengthen goal” (31%), and “stay away from venues” (30%). Several strategy groups were selected by fewer than 10% of participants, including “keep to budget,” “build momentum,” and “impose rewards and consequences.” Two strategies designed for in-venue use—“control cash in venue” and “slow down bets”—were also selected infrequently.

**Table 2:** Intervention strategy groups (*n* = 174)

Strategy Group (*n* = 25)	Mean	SD	Median	IQR 25%	IQR 75%	Total	Percent of participants
Avoidance							
Stay away from venues	1.86	1.73	1	1	2	78	30%
Financial management							
Keep to budget	2.25	1.26	2	1	3	9	3%
Limit cash	2.40	2.30	2	1	3	115	34%
Reduce cash in hand	1.41	0.67	1	1	2	31	16%
Maintaining momentum							
Build commitment	1.88	1.43	1	1	2	60	23%
Build momentum	1.29	0.76	1	1	1	9	5%
Know reason for change	1.20	0.41	1	1	1	30	18%
Strengthen goal	1.80	1.41	1	1	2	79	31%
Managing emotions							
Deal with emotions	1.38	0.71	1	1	2	33	17%
Rewards							
Grab a treat	1.89	1.65	1	1	2	100	38%
Impose rewards and consequences	1.09	0.30	1	1	1	12	8%
Substitution activities							
Do something else	3.67	5.13	2	1	4	305	59%
Swap gambling	1.13	0.34	1	1	1	18	11%
Social support							
Seek professional support	1.54	0.69	1	1	2	43	20%
Talk to someone	2.24	3.29	1	1	2	65	21%
Staying in control while gambling							
Control cash in venue	1.22	0.44	1	1	1	11	6%
Increase self-control in venue	1.52	0.80	1	1	2	41	19%
Know when to walk away	1.96	1.43	1	1	2	53	19%
Slow down the bets	1.00	0.00	1	1	1	4	3%
Pre-pokies prep	1.56	1.36	1	1	1	25	11%
Stress management							
Reduce stress	1.79	1.48	1	1	2	25	10%
Support good health	1.20	0.56	1	1	1	18	10%
Urge management							
Control gambling urges	1.64	0.89	1	1	2	72	31%
Know tricks pokies play	1.59	0.80	1	1	2	27	12%
Reduce gambling thoughts	1.52	0.78	1	1	2	44	20%

### Gambling habit hacker acceptability

#### Subjective quality and perceived impact

The Subjective Quality subscale had a mean score of 3.1 (SD = 0.8), with 61.0% of participants rating it as acceptable. The item assessing likelihood of recommending the app to others had a mean of 3.4 (SD = 1.2), with 75.2% indicating it met the threshold for acceptability. The item measuring likelihood of use in the next year had a mean of 3.3 (SD = 1.1), with 80.1% rating it as acceptable. The willingness to pay item had a mean of 2.1 (SD = 1.1), and 33.3% rated it as acceptable. The overall star rating item had a mean score of 3.6 (SD = 0.9), with 88.7% reporting it as acceptable. Participants also reported on the perceived impact of the app. Mean scores for these items ranged from 3.7 to 4.0. Awareness was rated with a mean of 4.0 (SD = 0.9), with 94.3% of participants indicating acceptability. Knowledge had a mean of 3.8 (SD = 1.0) and 92.2% acceptability. Attitudes toward gambling were rated at 3.8 (SD = 0.9), with 93.6% indicating acceptability. The item assessing intention to change gambling behaviour had a mean of 3.9 (SD = 1.0), with 90.1% rating it as acceptable. Help-seeking (M = 3.8, SD = 0.9) and behaviour change (M = 3.7, SD = 1.1) were rated as acceptable by 91.5% and 86.5% of participants, respectively. Descriptive statistics for individual items across both subscales are presented in Supplementary File S4.

#### Perceived helpfulness of additional app features

In the post-intervention survey, participants were asked to rate the perceived helpfulness of potential enhancements to *Gambling Habit Hacker* (see Table 3). Among the 141 participants who completed the survey, many indicated that support from various types of people would be helpful. These included a peer (defined as someone who has recovered from gambling issues; 86%), a qualified counsellor (81%), and an e-coach trained to support aspects of the program (80%). Participants were also asked about preferred support modalities. Chat-based support was most frequently selected (79%), followed by text messaging (73%), phone (67%), email (66%), and video conferencing (57%). In-person support was also rated as helpful by 65% of participants.

Regarding potential app features, the option to access the program at any time (i.e., a pull-based feature) was most frequently endorsed as helpful (87%). Several other features were also commonly rated as somewhat to very helpful: receiving motivational messages (83%), receiving graphical or other feedback on gambling behaviour (80%), saving favourite activities (79%), accessing a virtual AI coach (76%), receiving in-app rewards such as badges or points (74%), and participating in an online discussion board (73%).

**Table 3:** Perceived helpfulness of additional app features (*n* = 141)

	Not at all helpful	Slightly helpful	Somewhat helpful	Moderately helpful	Very helpful
**Potential qualifications of support**					
Qualified counsellor	4 (3%)	23 (16%)	40 (28%)	38 (27%)	36 (26%)
Peer	4 (3%)	16 (11%)	35 (25%)	40 (28%)	46 (33%)
E-coach	7 (5%)	21 (15%)	40 (28%)	34 (24%)	39 (28%)
**Potential modality of support**					
In person	20 (14%)	29 (21%)	25 (18%)	22 (16%)	45 (32%)
Video conferencing	25 (18%)	35 (25%)	22 (16%)	34 (24%)	25 (18%)
Telephone	20 (14%)	27 (19%)	27 (19%)	36 (25%)	31 (22%)
Email	18 (13%)	30 (21%)	35 (25%)	33 (23%)	25 (18%)
Chat	8 (6%)	21 (15%)	33 (23%)	33 (23%)	46 (33%)
Text messaging	10 (7%)	28 (20%)	34 (24%)	36 (26%)	33 (23%)
**Potential new app features**					
Peer Forum	9 (6%)	29 (21%)	34 (24%)	30 (21%)	39 (28%)
Access anytime	3 (2%)	16 (11%)	31 (22%)	37 (26%)	54 (38%)
Motivational messages	5 (4%)	19 (14%)	36 (26%)	42 (30%)	39 (28%)
Virtual computer coach	11 (8%)	23 (16%)	39 (28%)	35 (25%)	33 (23%)
Gambling feedback	7 (5%)	21 (15%)	26 (18%)	41 (29%)	46 (33%)
In-app rewards	12 (9%)	24 (17%)	24 (17%)	38 (27%)	43 (31%)
Saving favourites	10 (7%)	19 (14%)	36 (26%)	37 (26%)	39 (28%)

#### JITAI protocol preferences

Participants were asked to provide feedback on their JITAI preferences in terms of the EMA and program duration (*n* = 141). The highest endorsement was two EMAs (*n* = 54, 38%), followed by three EMAs (*n* = 40, 28%), one EMA (*n* = 37, 26%), four EMAs (*n* = 7, 5%) and five or more EMAs per day (*n* = 3, 2%). The preferred length of the program varied across the sample. One-third (*n* = 47, 33%) indicated a preferred program length of one month, but the majority wanted a longer program. Specifically, 2 months (*n* = 18, 13%), 3–4 months (*n* = 26, 18%), 5–6 months (*n* = 11, 8%), and more than six months (*n* = 20, 14%). In contrast, 17 participants (12%) indicated a preference for a 2-week program duration, and two participants (1%) indicated a preference for a 1-week program duration.

Participants overwhelmingly indicated a preference for a hybrid app that combined both pull and push features (*n* = 106, 75%), with fewer indicating a preference for a more traditional pull only approach (*n* = 18, 13%) or push only approach (*n* = 17, 12%). During the trial period, the app operated as “push” only during the 28-day MRT, meaning participants can check in via the 3 daily EMA push notifications, and “pull” only in the six-month follow-up period.

### Semi-structured interviews

Thematic analysis identified six major themes: expectations of the app and reasons for use; goal setting and implementation; EMA schedules and monitoring; engagement with intervention content; perceived usefulness of the content; and integration with other treatment and support options.

#### Expectations and reasons for use

Initial interest in *Gambling Habit Hacker* reflected varying levels of readiness to change among participants. Those in the early stages of considering change often had unclear expectations but were looking for easy-to-use tools that did not require professional oversight. Some came across the app through sponsored social media advertisements, while others were actively searching for self-help resources. One participant saw the app as a way to find out how much they really gambled—an insight-seeking approach that was interesting given they had already recognised their gambling as problematic and had been trying to change. Similarly, three participants who had been struggling with gambling for some time viewed the app primarily as a tool for setting goals and monitoring progress, rather than as a form of treatment.

For those already taking steps to change their gambling, the app was seen as a self-monitoring support tool—useful for tracking gambling behaviour, spending, and maintaining accountability. Several participants also viewed it as helpful in changing their thinking about gambling, manage responses to triggers and urges, and shift attitudes through greater self-awareness and insight.

*I was looking for something that was going to give me support that was going to check up on me at least once a day.* (Male, 24, moderate app use, mild severity)

*I found my whole mood lifted after about two weeks. I guess the app made me realize how much gambling had affected me.* (Female, 55, high use, moderate severity)

For participants who had previously received counselling, the app was seen as a convenient way to access support without needing to meet someone in person. One participant, who was experiencing severe and long-term gambling problems, used the app while waiting for a residential placement and was actively seeking immediate treatment. However, the app proved unsuitable for this individual, as they found it difficult to engage with the goals and intervention content. This highlights the importance of providing clear guidance about who the app is most appropriate for. Integrating a triage or screening process at the outset could help users make more informed choices. In this case, a different app designed for individuals with more severe gambling issues may have been a better fit.

#### Goal setting and goal implementation

The experience of setting gambling goals was varied and complex, reflecting both the challenges and the strategic adjustments required to manage gambling expenditure. Many participants found the idea of planning and setting specific gambling goals to be counterintuitive, particularly given their tendency toward impulsive behaviour. One participant described the difficulty of reconciling their immediate, reactive urges with the long-term planning approach encouraged by the app, noting that setting goals for something as spontaneous as gambling felt unnatural. Others shared that gambling itself felt at odds with their personal values, creating an internal conflict when trying to engage with the goal-setting process. For some, setting a goal of zero gambling was more straightforward, but for those aiming to reduce rather than eliminate gambling, following through with intended gambling often led to cognitive dissonance.

*It changed the way I think about gambling… the driving force was just the fact that I set a goal.* (Male, 24, moderate use, mild severity)

*It was a little bit challenging only because like when you are in a mindset you don’t necessarily want to reflect on how much you have spent*. (Male, 27, high use, moderate severity)

*Supposed to stop completely*,* but the app sort of encouraged you to make realistic plans*,* which I personally didn’t agree with.* (Male, 42, low use, extreme severity)

Participants with less severe gambling problems reported that setting expenditure goals was helpful. For those interested in self-monitoring and tracking their spending, the idea of setting a goal was novel and engaging—something many had not previously considered. Several participants also expressed a desire to set broader goals related to work, stress management, exercise, and general well-being, and suggested linking these goals to the EMA protocol and planning interventions within the app.

The relationship between goal setting and goal implementation varied across participants. Some were highly aware of their intentions and adjusted their weekly expenditure to remain aligned with a broader monthly goal. This approach allowed for flexibility—participants could accommodate social events by spending more at certain times and compensating by cutting back at others. However, not all experiences were positive. One participant reported exceeding their expenditure goal almost immediately, which led to negative feelings. Others described selecting intended gambling days at random during goal setting, which made those goals feel arbitrary and less effective. Multiple participants recommended incorporating real-time feedback into the app to better support goal adherence. They suggested features that could show progress against intentions—such as daily expenditure compared to set goals—and provide immediate feedback to help the person stay on track.

#### EMA schedules and monitoring

Participants had mixed views about the frequency, timing, and content of the EMA protocol. Those using the app for self-monitoring generally felt that the EMA prompts were well-timed and manageable. They described them as quick, easy to answer, and not intrusive. One participant noted that the constant presence of the app was helpful, providing a sense of support and helping them stay on track. In contrast, others found the EMAs too frequent, repetitive, and tedious. Some suggested that while the timing should remain predictable, the questions could be more varied or focused on building motivation to maintain engagement. Others proposed varying the items, adjusting the timing, or changing the length of the EMAs to reduce monotony and improve user experience.

*Because I was reminded on a daily basis*,* I was more thoughtful about my gambling.* (Female, 32, low use, moderate severity)

*I think once a day was even too much because my problems were not during the week at all. My problem was weekends.* (Male, 55, high use, moderate severity)

*If there was more variation in questions or you mixed them up and had them in a different order*,* it could have been a bit more productive.* (Female 52, high use, mild severity)

Participants expressed a desire for greater flexibility in the EMA protocol, particularly regarding the timing, frequency, and content of prompts. Many felt that customisable alerts and reminders would better accommodate individual schedules, needs, and levels of gambling risk. Several noted that there were times when checking in was not possible — especially for those working in retail, hospitality, or other environments where mobile phone use is restricted. Some suggested that linking EMA availability to their calendar would provide more control over when prompts occurred. Additionally, participants reported feeling more vulnerable late at night, yet the final EMA prompt typically arrived in the early evening. One participant observed that the prompts often came when they were not needed but were absent when support would have been most useful.

Regarding frequency, some participants preferred to set the EMA schedule themselves, aligning prompts with typical gambling times. For those seeking help with urge management, one or two prompts per day were considered sufficient, and there was a view that none were needed on days when they were not at risk of gambling. Some participants reported that receiving too many reminders triggered gambling thoughts, highlighting the importance of maintaining a careful balance—tools designed to detect vulnerability should not inadvertently increase it.

Use of the EMA protocol in gambling venues was reported to be infrequent. Participants noted that while gambling, they often ignored or did not notice their phone; some turned their phones off entirely or could not hear alerts due to venue noise. Gambling in the evening also meant that they forgot about the protocol when consuming alcohol or socialising while gambling.

#### Engagement with intervention content

Participants suggested that the planning function could be better integrated into the app to reflect a more natural way of setting and implementing plans. Several participants recommended that each EMA include a recap of previous plans and allow users to assess whether those plans needed to be implemented or adjusted. Participants also noted that while they often created good plans, these were no longer available afterward; they suggested it would be helpful for the app to store plans for future use. Features like a favourites option or the ability to rank plans for different high-risk EMA situations were considered likely to improve usability.

*I would like to access those plans after I do them. It would be go good to go back and actually access those instead of just trying to recreate them a couple of weeks later.* (Male, 27, high app use, moderate gambling severity)

At the end of each intervention, participants were presented with a visualization activity, which asked them to imagine themselves successfully carrying out their intended actions. Responses to this activity were mixed, often due to time constraints. Some participants recommended offering visualizations of varying lengths to match individual needs and available time, while others suggested providing different delivery formats, such as audio or visual content. Tailoring the visualization activities to specific plans was also suggested.

The app featured extensive lived experience stories as part of the planning intervention. Participants valued these stories, noting that they were relatable, boosted confidence, and offered a sense of hope. Several participants proposed adding peer support features, such as a chat or forum with other app users. Others suggested making the stories more engaging by including real faces or voice recordings, as they found listening to real people (rather than a narrator’s voice) more meaningful, while the current animation approach felt depersonalized for some.

*Even just a recording of someone talking would make it feel more personal — a sound bite sharing their story. The cartoon face felt less engaging*,* and it occurred to me that hearing someone’s voice would be a nice touch.* (Female, 32, low app use, moderate gambling severity)

Some also commented that the app’s look and feel was somewhat dull and lacked the vibrancy of gambling apps, recommending that the design be tailored to enhance user experience, possibly with versions adapted for online versus land-based gambling contexts. Incorporating interactive elements, such as acted-out videos or scenarios, was seen as another way to make the experience more engaging.

#### Usefulness of intervention content

The underlying theory of *Gambling Habit Hacker* was to support skill development, particularly in self-awareness, self-efficacy, problem-solving, and planning. Most participants reported increased self-awareness of high-risk gambling situations, fluctuations in stress and emotions, and the broader everyday consequences of gambling. For example, one participant noted that the app helped them recognise feelings of stress and the need to address it, while another said the app detected their fatigue, which they had not previously noticed. Two participants reported difficulty selecting the right strategy, especially when feeling stressed, and suggested that a “pick for me” option could have been helpful during such times.

The app also promoted personal growth by reminding participants of their intrinsic qualities and strengths. One participant reflected on the value of being reminded of their strengths, especially when feeling disappointed about their gambling behaviour. However, some participants found the introspective aspects challenging, particularly when asked to identify their strengths. One participant described difficulty in recognising their own strengths, highlighting the need for program adjustments to better support users in identifying and applying their strengths effectively.

*When you’re in the throes of gambling problems*,* you tend to forget that you actually are good at a lot of things and you’re a good person.* (Female, 55, high use, moderate severity)

Participants were prompted to identify obstacles to implementing their plans. Many reported that the program helped them develop greater self-awareness, enabling them to recognise specific high-risk situations and triggers that intensified their gambling urges. For example, one participant highlighted the effectiveness of leaving their phone at home to avoid impulsive gambling during walks. However, participants noted that simply recognising obstacles was often not enough to overcome them. They suggested that it would be helpful for the app to follow up on strategy use during subsequent check-ins. Timely follow-ups on the effectiveness of chosen strategies would allow users to adjust their plans as needed, particularly in response to external factors like weather, which could disrupt planned activities.

#### Support and integration with other treatment options

The app played a significant role in fostering a sense of accountability among participants. Its built-in mechanisms supported goal setting and encouraged meaningful changes in gambling behaviour. Some participants described the app as more of a supportive companion than a strict accountability tool, highlighting the value of its relational aspects. For these individuals, the app offered comfort and encouragement, not just monitoring, which made the experience feel more personal and less punitive.

*It was kind of like my buddy*,* especially in the first couple of weeks*,* just coming in and checking on me. It would’ve been really good if they also checked in on me at night.* (Male, 55, high app use, moderate gambling severity)

While the app provided a sense of support, some participants felt that being accountable to a real person would be even more effective. The app included strategies for building social connections, repairing relationships, and seeking social support. One participant involved their partner while using the app, while another noted that discussing activities with their partner added a helpful layer of external accountability. Beyond personal relationships, several participants discussed the potential for integrating the app into professional support settings. Some saw the app as a useful complement to counselling, while others suggested it would be even more beneficial if therapists could review app data or incorporate its use into therapy sessions.

## Discussion

This paper reports on the acceptability of a JITAI designed to support adherence to gambling expenditure goals, with the broader aim of reducing gambling over the longer term. It is the first study to examine the acceptability of a JITAI that integrates goal setting, action and coping planning, implementation support, and self-monitoring through a smartphone application for individuals experiencing gambling problems. Key findings are drawn from app use and engagement metrics, app acceptability measures, and semi-structured interviews. Together, the quantitative and qualitative data provide new insights into the BCTs embedded in the intervention, as well as the delivery and engagement mechanisms relevant to JITAI design. Intervention fidelity was evaluated based on adherence to the EMA protocol, strategy selection, and action plan completion. In addition, several areas for intervention optimisation were identified.

### App functionality, content and EMA protocol

App ratings exceeded the minimum threshold for acceptability and were consistent with findings from previous research, including evaluations of publicly available apps [11, 16]. However, low compliance with the EMA protocol suggests there is scope to improve engagement. Participants expressed strong support for a hybrid delivery model, in which app use is complemented by support from a qualified counsellor, peer, or coach. No single mode of delivery was clearly preferred, with participants indicating that a range of formats—including in-person, online, phone, or text message—could be acceptable. *Gambling Habit Hacker* was designed for individuals with less severe gambling problems. While approximately half the sample reported low to moderate symptom severity on the G-SAS, a notable proportion had severe gambling symptoms. To better reach the intended audience, future efforts may need to explore targeted marketing and promotional strategies aimed at individuals experiencing less severe gambling-related harm.

Participants generally reported that the app was satisfactory in terms of its look, feel, and interactivity, but noted that there was room for improvement. Qualitative feedback identified several opportunities to enhance users’ personal connection with the app, particularly in relation to intervention delivery. The app included a substantial amount of text-based content, comprising short segments that described how others had implemented each of the 120 strategies. This implementation support was drawn from lived experience and synthesised across several studies [25–27, 29, 30]. Participants expressed strong appreciation for this content and suggested that it could play a more central role in the app. For example, many indicated that implementation support would be more engaging if delivered via video or audio, enabling them to hear directly from individuals who had attempted the strategies. Others proposed a more personalised approach, such as recording their own plans, which could then be replayed when the EMA identified a moment of need. These suggestions highlight the value of lived experience and the potential of storytelling as a means of support. Future research could examine whether strategies are more likely to be adopted when delivered via text, clinician guidance, or a lived experience voice—transforming the app into a platform that combines evidence-based techniques with the authenticity of lived narratives.

While the intervention content was generally well-rated, some participants found the tailored strategies repetitive. Nonetheless, participants reported improvements in knowledge, awareness, and attitudes toward gambling, alongside increased intention to reduce expenditure and seek help when needed. Qualitative data further indicated gains in self-awareness, problem-solving, recognition of personal barriers, and the capacity to enact plans. Participants also described enhanced confidence and competence in managing gambling-related urges. These findings align with the trial’s quantitative outcomes [37], which showed significant improvements in self-efficacy, well-being, and psychological distress, along with reductions in gambling symptoms. The perceived repetitiveness of some content may be attributable to the uniform formatting of the intervention components, which offered limited variation in presentation across tailored interventions.

There was discrepancy between the quantitative and qualitative findings regarding the frequency, timing, and content of the EMA protocol. Quantitative data indicated that while some participants wanted more EMAs, most preferred fewer. Similarly, although many participants expressed a desire for a longer program duration of two months or more, others preferred a shorter period of less than one month. Qualitative data helped explain these differences, suggesting they were largely shaped by participants’ varying expectations and reasons for using the app. Participants who used the app primarily for goal setting and self-monitoring generally welcomed regular check-ins but preferred that these evolve over time. For example, if a participant was no longer experiencing gambling urges, they preferred the app to stop asking about urges and instead focus on maintaining motivation or other relevant goals. In contrast, participants who engaged with the app primarily for its intervention components preferred receiving EMAs during periods of increased vulnerability—such as times they typically gambled or thought about gambling, often on weekends or late at night. These participants found it burdensome to be asked about gambling during times when it was not relevant or when they did not want to be reminded of it. Preferences for the overall duration of the program also varied. Those who used the app to track their gambling behaviour tended to favour a longer timeframe, while those focused on urge management preferred fewer, but more precisely timed, check-ins. These findings highlight the need to account for variability in user preferences regarding EMA frequency and program length. Personalising the intervention to reflect these differences—particularly as needs evolve over the course of treatment—may enhance engagement and acceptability.

### Goal setting, planning and implementation support

To our knowledge, this is the first study to prompt individuals with gambling problems to set daily expenditure goals, track progress toward these goals, and self-monitor factors that could influence their goal intentions. Technically, setting a specific daily goal was not difficult for participants, marking an improvement from prior usability testing, in which goal setting was identified as the most significant technical barrier [22]. Several participants described the goal-setting process as both interesting and helpful in increasing self-awareness of their intended versus actual gambling expenditure. The Timeline FollowForward used in this study was adapted from the Timeline FollowBack [48] and previously piloted in a study on video gaming [32]. However, it has not yet been validated for use in gambling treatment settings. Given participants’ positive reception of this approach, future research could examine its predictive utility as a standalone intervention and in combination with goal monitoring features.

Despite its usefulness for many, some participants struggled with the concept of planning to gamble—even when their goals involved reduction rather than abstinence. Barriers to planning included feelings of guilt and the belief that gambling was impulsive, not something to be scheduled. Quantitative data showed that most app users had moderate symptom severity, with approximately 20% experiencing extreme symptoms. Among two interview participants in this category, one expressed that planning to gamble felt morally wrong, while the other reported increased self-awareness from setting goals, noting it was their first time tracking expenditure. These insights point to the need for further investigation into the acceptability of the Timeline FollowForward across the full spectrum of gambling severity.

Although goal setting and monitoring can be implemented without a JITAI, participants who used the app primarily for self-monitoring reported that the EMA protocol helped them track shifting emotions and thoughts about gambling. Many described a sense of accountability to the app, which they found motivating and helpful in adhering to their goals. The intervention design was informed by self-determination theory [31], aiming to support autonomy, relatedness, and competence. This was reflected in the use of motivational interviewing principles, non-judgemental language, and the ability for users to set their own goals—including goals that involved controlled gambling.

Participants also expressed a desire to track a broader range of goals, such as resisting urges or monitoring gambling-related thoughts. These suggestions imply that the EMA protocol may need to be adjusted to support multiple concurrent goals. Such feedback also opens the possibility of developing an extended version of *Gambling Habit Hacker*, focused specifically on multi-goal support through tailored EMA delivery. Prior research on web-based interventions suggests that even simple self-monitoring tools—offered with minimal support—can be effective in reducing gambling harm [49]. While other mHealth apps include features such as goal setting and self-monitoring, few have been formally evaluated [8] and some rely on passive delivery through messaging platforms [50]. Notably, self-monitoring is a BCT consistently associated with improved outcomes in addiction treatment [51].

Quantitative findings also showed that participants created more than 1,300 action and coping plans using pre-specified implementation support. The most frequently selected plans involved substitution activities, designed to address vulnerabilities such as urges or negative emotions. While traditional implementation planning typically involves a single session conducted in advance of a triggering situation [23, 34] research suggests that people with gambling problems may struggle to anticipate barriers—even when prompted [28].

In this study, participants developed an average of eight different plans each—far more than is typical. The intervention was intentionally designed to support the creation of multiple plans over time, acknowledging that new barriers often emerge during the change process [22]. However, some participants described the process as burdensome, particularly when prompted to recreate the same plan multiple times for recurring vulnerabilities. A key suggestion was the ability to save and reuse plans—a feature not currently available in the app. Participants also proposed developing initial plans during registration, which could then be refined and deployed as needed via the EMA protocol.

The intervention also incorporated a proximity-based EMA and response protocol, designed to identify gambling risk based on time, day, and context. This feature prioritised in-venue risk by recommending specific strategies when gambling proximity was detected. However, quantitative data indicated that in-venue strategies were rarely selected. This finding aligns with previous research showing that most strategies are implemented before gambling sessions—for example, leaving cards at home or limiting access to cash [28].

The low uptake of in-venue strategies suggests that few EMAs were completed during actual gambling episodes. Qualitative feedback indicated that this was likely due to participants turning off their phones in venues or being unable to hear or respond to the app due to environmental noise. Findings from the associated outcomes study [37] showed that participants who did gamble tended to exceed their planned limits, although they generally spent less than they would have otherwise. This may reflect the app’s limited effectiveness in venue settings—either because the intervention was not delivered or because users deliberately disengaged during gambling episodes.

These results suggest that planning in advance may be essential to support in-venue behaviour change. One potential solution could involve delivering a personalised in-venue plan prepared on the day a gambling episode is anticipated. However, the findings also indicate that in-venue interventions may require an entirely different approach. Once individuals enter a gambling venue, they may become effectively unreachable via digital tools. This highlights the importance of broader structural and policy-level interventions, such as mandatory pre-commitment systems (e.g., card-based gambling), and reforms to gambling products and environments. Modifying features that encourage prolonged gambling— such as jackpots, free spins, or in-play betting—may be necessary to complement app-based behavioural strategies and reduce gambling harm.

### Study strengths and limitations

This study has several strengths, including its mixed-methods design, which combined app usage metrics from a large quantitative sample with a small sub-sample of in-depth qualitative interviews; however, the small qualitative sample size should be acknowledged as a limitation and highlights the need for future research with larger samples to confirm these findings. This approach yielded initial insights into how and why people engaged with the app, enabling the intervention to be further tailored to the needs and preferences of the target population. However, several limitations should be considered when interpreting the findings. First, data extraction from the app was complex due to the absence of a user-friendly back-end infrastructure. As a result, substantial effort was required to clean and organise the quantitative data, and not all components of the program could be reliably linked—for example, EMA tailoring variables were not consistently matched with subsequent plan selections.

Second, compliance with the EMA protocol—particularly with regard to entering gambling expenditure—was lower than anticipated. Although participants were prompted to enter this data during subsequent check-ins, some expenditure entries remained missing. This suggests potential issues with receptivity and participant burden, highlighting the need to explore strategies that enhance engagement and reduce fatigue. Given that participants expressed differing preferences for EMA delivery, expenditure tracking may be more effective if entered directly into the app at the time of gambling, or alternatively, through a once-daily summary entry, either at the end of the day or the following morning [52]. Qualitative feedback also highlighted concerns about the repetitiveness of EMA items. Randomising the presentation of questions—rather than asking the same items in the same order at every time point—may increase participant engagement and improve compliance. These findings raise important questions about how much and how often participants are willing to provide self-reported data, suggesting that future research should also explore more passive forms of data collection.

Finally, interviews conducted after the intervention revealed that participants with lower app usage had difficulty recalling specific app features, potentially limiting the depth of their responses. This recall bias likely led participants to focus only on the most salient aspects of their experience. One strategy to address this limitation is cognitive interviewing, where participants verbalise their thoughts while actively engaging with the app [53]. However, this approach may not capture perceptions of how the app integrates into everyday life. An alternative could involve real-time or near-real-time data collection during the intervention period—such as brief daily or weekly check-ins or EMA-based reflections designed specifically for interviewees.

### Optimisation implications

Quantitative and qualitative findings indicated that participants were interested in both peer and professional support. Qualitative data revealed that some participants were already using the app in conjunction with support from a specialist gambling counsellor. Several users described the app as a complement to psychological treatment and suggested that integrating the app with professional support—such as having therapists review app data—could further enhance its utility. Participants also highlighted the need for greater integration of the app into daily life, including the involvement of significant others and social networks. A version of the app designed for use at home as part of a broader treatment plan could be beneficial. However, caution is warranted when recommending this approach to individuals with more severe gambling problems or those at risk of relapse. In such cases, a relapse-specific intervention like *GamblingLess: In-the-Moment* [21, 47] - a JITAI grounded in cognitive-behavioural therapy and designed for active gambling and relapse prevention—may be more appropriate.

For individuals with less severe problems, blended approaches that combine clinician input with digital tools may improve engagement, particularly when support is provided via a single session, online format, or phone-based counselling [54]. The EMA protocol could be enhanced to generate a summary of EMA responses and intervention activities, which could then be reviewed in counselling sessions to facilitate reflection and discussion. Future research may also explore new formats for plan development, including options for peer or professional support and virtual in-app guidance. Incorporating human support in this way may improve compliance with EMA protocols, as existing evidence suggests human involvement increases engagement with mHealth interventions [55].

*Gambling Habit Hacker* operated solely as a push intervention, meaning that users could not initiate support independently of the EMA schedule. Some participants reported experiencing times of heightened vulnerability—such as late at night or between check-ins—when they would have benefited from immediate support that was not available. A hybrid model combining push and pull features may be optimal, allowing users to both receive scheduled prompts and access tailored intervention strategies on demand [13, 56].

In addition to these optimisation considerations, it is important to address engagement with the app itself. Despite the app’s promise, compliance with the EMA protocol was modest, with an average completion rate of 30% across the 28-day period and notably lower completion among male participants. This finding aligns with prior research showing that men are typically harder to engage and retain in gambling interventions [57] and underscores the need to develop targeted strategies to improve engagement across subgroups. Addressing compliance is crucial for optimising the effectiveness of JITAI interventions, as sustained user engagement is key to maximising their therapeutic impact. Future research should explore adaptive engagement strategies, including personalised reminders, flexible scheduling, and human support features, to enhance compliance and ensure more consistent data capture.

*Gambling Habit Hacker* represents a novel approach to delivering a low-intensity, non-CBT-based intervention. While the delivery mechanism is innovative, the core components—goal setting and implementation planning—are supported by a strong evidence base [35, 36, 58, 59]. A key challenge in self-regulation is the tendency to override long-term goals in favour of immediate impulses, and failure to consider the long-term consequences of behaviour [60]. Although some participants reported a desire to reset their goals after exceeding their intended expenditure, self-regulation theory suggests the focus should be on adjusting behaviour rather than altering the goal itself. The app could be strengthened by providing more explicit support for goal self-efficacy. Although relevant strategies were included in the app, they were rarely triggered due to their placement low in the tailoring hierarchy [37].

Findings from the outcome study [37] showed that EMA entries frequently included references to harms such as interpersonal conflict, negative emotions, and financial stress. While recent public health initiatives have increasingly focused on gambling harm reduction, these efforts have typically targeted attitude change or reductions in expenditure—similar to the current app’s emphasis. Notably, *Gambling Habit Hacker* also included non-gambling support strategies, such as assertiveness training, stress reduction, social reconnection, and debt management. This content could be further developed to directly address proximal stressors and the harms that may persist after gambling behaviours have changed. Ideally, such an intervention would be targeted toward individuals experiencing lower levels of harm and without co-occurring mental health or addiction conditions.

## Conclusion

The *Gambling Habit Hacker* app represents a promising advancement in the delivery of low-intensity, evidence-based interventions for reducing gambling-related harm. This mixed-methods study demonstrated strong support for the app’s content and overall purpose, with participants valuing the tailored intervention strategies provided. At the same time, the study identified several areas for improvement, particularly in enhancing delivery mechanisms, streamlining the planning process, and increasing user engagement through a combination of push- and pull-based features. Incorporating professional or peer support, and addressing proximal stressors, may further strengthen the app’s effectiveness—particularly for individuals experiencing less severe gambling problems. Future research should prioritise optimising the app to accommodate diverse user needs, enhancing the tailoring of interventions to reinforce goal commitment, and exploring the role of human support in improving engagement and adherence. The insights gained from this study offer a valuable foundation for refining *Gambling Habit Hacker* and similar digital interventions. By advancing their usability, personalisation, and support features, such tools can contribute meaningfully to more effective and accessible support for individuals seeking to manage their gambling behaviours.

## Electronic supplementary material

Below is the link to the electronic supplementary material.

Supplementary Material 1

## Data Availability

Anonymised data is available by contacting the corresponding author.
