# Supplementary material for: Acceptability of the content and functionality of a just-in-time adaptive intervention for gambling problems: a mixed-methods evaluation of gambling habit hacker
Source: Addict Sci Clin Pract. 2025 Jun 5;20:46. doi: 10.1186/s13722-025-00573-y (PMC12139257; doi:10.1186/s13722-025-00573-y)
Supplement: Supplementary file 1 — Supplementary Material 1 [file 13722_2025_573_MOESM1_ESM.docx]

Supplementary File S1. Socio-demographic characteristics

| Socio-demographic characteristic | | M | SD | Median | n | % | 95% CI |
| --- | --- | --- | --- | --- | --- | --- | --- |
| Age (years) | | 35.7 | 11.7 | 34.5 |  |  | (34.0, 37.5) |
| Gender | |  |  |  |  |  |  |
|  | Male |  |  |  | 119 | 68.4% | (61.1, 74.9) |
|  | Female |  |  |  | 54 | 31.0% | (24.6, 38.3) |
|  | Unspecified gender |  |  |  | 1 | 0.57% | (0.08, 4.0) |
| Ethnicity | |  |  |  |  |  |  |
|  | Australian |  |  |  | 130 | 74.7% | (67.7, 80.7) |
|  | Australian-mixed |  |  |  | 23 | 13.2% | (8.9, 19.2) |
|  | Other |  |  |  | 21 | 12.1% | (8.0, 17.9) |
| State of residence* | |  |  |  |  |  |  |
|  | New South Wales |  |  |  | 70 | 53.4% | (44.8, 61.9) |
|  | Victoria |  |  |  | 26 | 19.9% | (13.8, 27.6) |
|  | Queensland |  |  |  | 18 | 13.7% | (8.8, 20.8) |
|  | South Australia |  |  |  | 7 | 5.3% | (2.6, 10.9) |
|  | Western Australia |  |  |  | 5 | 3.8% | (1.6, 8.9) |
|  | Tasmania |  |  |  | 5 | 3.8% | (1.6, 8.9) |
| Annual personal gross income (AUD$) | |  |  |  |  |  |  |
|  | Less than $25,000 |  |  |  | 18 | 10.3% | (6.6, 15.9) |
|  | $25,000 to $49,999 |  |  |  | 30 | 17.2% | (12.3, 23.6) |
|  | $50,000 to $74,999 |  |  |  | 57 | 32.8% | (26.2, 40.1) |
|  | $75,000 to $99,999 |  |  |  | 33 | 19.0% | (13.8, 25.5) |
|  | $100,000 to $124,999 |  |  |  | 16 | 9.2% | (5.7, 14.5) |
|  | $125,000 to $149,999 |  |  |  | 14 | 8.1% | (4.8, 13.2) |
|  | $150,000 to $174,999 |  |  |  | 3 | 1.7% | (0.55, 5.3) |
|  | $175,000 to $199,999 |  |  |  | 0 |  |  |
|  | $200,000 or more |  |  |  | 3 | 1.7% | (0.55, 5.3) |
| SEIFA (IRSAD)^a *^ | | 989.2 | 76.0 | 985.0 |  |  | (976.1, 1002.3) |

a SEIFA: Socio-Economic Indexes for Areas; IRSAD=Index of Relative Socio-Economic Advantage and Disadvantage; * missing = 24.7% n=174 (pre-intervention analytic sample)

Supplementary File S2. Interview participant characteristics

| ID | Gender, Age | G-SAS gambling symptom severity | Problem gambling activity | Intended $ | $ goal | App use |
| --- | --- | --- | --- | --- | --- | --- |
| GHH01 | Male, 27 | moderate | EGM | $400 | 87% decrease | high |
| GHH02 | Female, 20 | mild | Sports, Numbers | $15 | Stay same | moderate |
| GHH03 | Male, 50 | severe | Sports, wagering | $300 | 85% decrease | moderate |
| GHH04 | Female, 55 | moderate | wagering | $140 | Stay same | high |
| GHH05 | Male, 32 | mild | Numbers, wagering | 0 | Stay same | low |
| GHH06 | Female, 32 | moderate | EGM, wagering | $740 | 32% decrease | low |
| GHH07 | Male, 52 | mild | EGM, Sports, tables, Numbers | $350 | Stay same | high |
| GHH08 | Male, 24 | mild | Sports, Numbers | $70 | 74% decrease | moderate |
| GHH09 | Male, 55 | moderate | EGM | $30 | 99% decrease | high |
| GHH10 | Female, 52 | mild | EGM | 0 | 100% decrease | high |
| GHH11 | Male, 42 | extreme | EGM | $450 | 80% decrease | low |

Low app use = 4-8 EMAs completed, moderate app use = 9-58 EMAs completed, high app use = 59+ EMAs completed.

Supplementary File S3. Timing of app use across the day and 28-day period

|  | | Mean | SD | Median | IQR 25% | IQR 75% |
| --- | --- | --- | --- | --- | --- | --- |
| Time of day | |  |  |  |  |  |
|  | Morning | 8.13 | 8.86 | 4 | 1 | 14 |
|  | Afternoon | 8.52 | 9.37 | 3 | 1 | 16 |
|  | Evening | 8.53 | 9.24 | 4 | 1 | 15 |
| Week of MRT period | |  |  |  |  |  |
|  | Week 1 | 8.39 | 6.90 | 6 | 2 | 15 |
|  | Week 2 | 6.03 | 7.03 | 2 | 0 | 12 |
|  | Week 3 | 5.38 | 7.04 | 1 | 0 | 12 |
|  | Week 4 | 5.39 | 7.26 | 0 | 0 | 11 |

Supplementary Table S4. Item-level descriptive statistics for the MARS Subjective Quality and Perceived Impact subscales (n=141)

| MARS Subjective Quality subscale | | | | | | | |
| --- | --- | --- | --- | --- | --- | --- | --- |
| Recommend to others^a^ | Not at all |  | Maybe |  | Definitely | Mean | SD |
|  | 6 (4%) | 29 (21%) | 45 (32%) | 32 (23%) | 29 (21%) | 3.35 | 1.15 |
| Next-year use^b^ | None | 1-2 | 3-10 | 10-50 | 50+ |  |  |
|  | 13 (9%) | 15 (11%) | 44 (31%) | 49 (35%) | 20 (14%) | 3.34 | 1.13 |
| Prepared to pay^c^ | Definitely not |  |  |  | Definitely yes |  |  |
|  | 49 (35%) | 45 (32%) | 33 (23%) | 7 (5%) | 7 (5%) | 2.13 | 1.10 |
| Overall star rating^d^ | * | ** | *** | **** | ***** |  |  |
|  | 3 (2%) | 13 (9%) | 42 (30%) | 63 (45%) | 20 (14%) | 3.60 | 0.92 |
| MARS Perceived Impact subscale^e^ | | | | | | | |
|  | Strongly disagree | Disagree | Neither agree nor disagree | Agree | Strongly agree |  |  |
| Awareness | 5 (4%) | 3 (2%) | 21 (15%) | 71 (50%) | 41 (29%) | 3.99 | 0.92 |
| Knowledge | 5 (4%) | 6 (4%) | 35 (25%) | 64 (45%) | 31 (22%) | 3.78 | 0.96 |
| Attitudes | 4 (3%) | 5 (4%) | 35 (25%) | 67 (48%) | 30 (21%) | 3.81 | 0.91 |
| Intention to change | 6 (4%) | 8 (6%) | 18 (13%) | 72 (51%) | 37 (26%) | 3.89 | 1.00 |
| Help-seeking | 4 (3%) | 8 (6%) | 33 (23%) | 66 (47%) | 30 (21%) | 3.78 | 0.94 |
| Behaviour change | 7 (5%) | 12 (9%) | 27 (19%) | 62 (44%) | 33 (23%) | 3.72 | 1.07 |

^a^ Response options from (1) Not at all (I would not recommend this app to anyone) to (5) Definitely (I would recommend this app to everyone)

^b^ Response options from (1) *None* to (5) *50+*

^c^ Response options from (1) *Definitely not* to (5) *Definitely yes*

^d^ Response options from (1) * One of the worst apps I’ve used to (5) ***** One of the best apps I’ve used

^e^ Response options from (1) Strongly disagree to (5) Strongly agree
